# Real‐World Efficacy and Systemic Immune Modulation Upon Tralokinumab Treatment in Atopic Dermatitis

**DOI:** 10.1002/clt2.70200

**Published:** 2026-09-06

**Authors:** Meslina Almaci, Philipp Globig, Davender Redhu, Margitta Worm

**Affiliations:** ^1^ Division of Allergy and Immunology Department of Dermatology, Venerology and Allergy Charité Universitätsmedizin Berlin Berlin Germany

**Keywords:** atopic dermatitis, biologics, IL‐13, T‐cells, tralokinumab

## Abstract

**Background:**

Tralokinumab, an interleukin‐13 (IL‐13) monoclonal antibody, has shown clinical efficacy in randomized trials for moderate‐to‐severe atopic dermatitis (AD). This study aims to evaluate clinical and immunological outcome upon tralokinumab treatment in a real‐world cohort of patients with moderate‐to‐severe AD.

**Methods:**

We recruited 81 AD patients receiving tralokinumab, performing clinical assessments (oScorad, IGA, BSA, DLQI, pruritus‐VAS) at baseline and months 3, 6, 9, and 12. For immunophenotyping (*n* = 25), PBMCs were collected at baseline and months 6/12. Peripheral T‐cell subsets, CLA, OX40, TFH, and cytokines (IL‐4, IL‐13, IL‐17A, IL‐10, IFNγ) were analyzed via multiparametric flow cytometry. Serum total/specific IgEs were measured by ImmunoCap and eosinophils by differential blood counts.

**Results:**

Tralokinumab treatment induced a continued clinical response: at 12 months, oScorad and BSA decreased by 60.8% and 78.8%. CLA^+^CD4^+^ and CD8^+^ T cells declined by 63.0% and 25.8%, and OX40^+^ CD4^+^/CD8^+^ T cells by 62.4% and 44.0%. CD4^+^ effector memory T cells increased 4.6‐fold, while TEMRA cells decreased 34.0%. CD4^+^ T cells expressing IL‐13, IL‐4, and IL‐17A declined by 70.8%, 72.8%, and 69.3%, while IFNγ^+^ and IL‐10^+^ CD4^+^ T cells increased by 62.0%. Total IgE decreased by 9.7%, while eosinophil counts remained stable.

**Conclusions:**

In real‐world practice, tralokinumab achieves robust clinical benefit and a rebalanced systemic immune pattern. Selective IL‐13 blockade reshapes T‐cell function, shifting the immune balance from Th2/Th17 dominance toward Th1/regulatory profiles, suggesting immunomodulatory effects consistent with disease modification. Future studies are needed to determine the long‐term stability of these immunological changes following treatment prolongation or interruption.

## 1 Introduction

Atopic dermatitis (AD) is a frequent, chronic inflammatory skin disease with a broad range of clinical manifestations, driven by complex underlying mechanisms [1]. It is marked by recurrent skin lesions and severe itching, which can result in painful excoriations [2]. AD impairs the quality of life, sleep, mental health and work productivity [3] in affected patients. Long‐term treatment is often necessary to maintain disease control and to prevent relapses [4].

The pathophysiology of AD is characterized by a complex interplay between barrier dysfunction and immune abnormalities. The activation of Th2 cells, with the production of cytokines like IL‐4, IL‐5 and IL‐13 play a key role in the pathophysiology of the disease [5, 6]. Among these, IL‐13 is a crucial driver of disease progression, contributing to the disruption of the skin barrier and the inflammatory process [7, 8]. Tralokinumab, an IgG4‐monoclonal antibody binds to IL‐13 and inhibits the interaction with its receptor. It was approved in 2021 for the treatment of moderate‐to‐severe AD [9]. Clinical trials have demonstrated its efficacy in improving clinical signs and patient‐reported outcomes, with a good safety profile [10, 11].

While several real‐world studies and case series evaluating tralokinumab have been published until now, data with larger patient cohorts and long‐term follow‐up periods are still limited [12, 13, 14, 15].

While the mechanism of selective IL‐13 blockade is well established, its broader impact on systemic T‐cell immunity remains still incompletely characterized.

Given the central role of IL‐13 in AD pathogenesis, selective IL‐13 blockade with tralokinumab provides an opportunity to investigate its impact on systemic immune responses. In this study, we investigated in a real‐world setting the clinical, but also peripheral T cell phenotypes in AD patients undergoing tralokinumab treatment during a 12‐month observation time.

## 2 Material and Methods

### 2.1 Study Design and Clinical Data Collection

**FIGURE 1:**
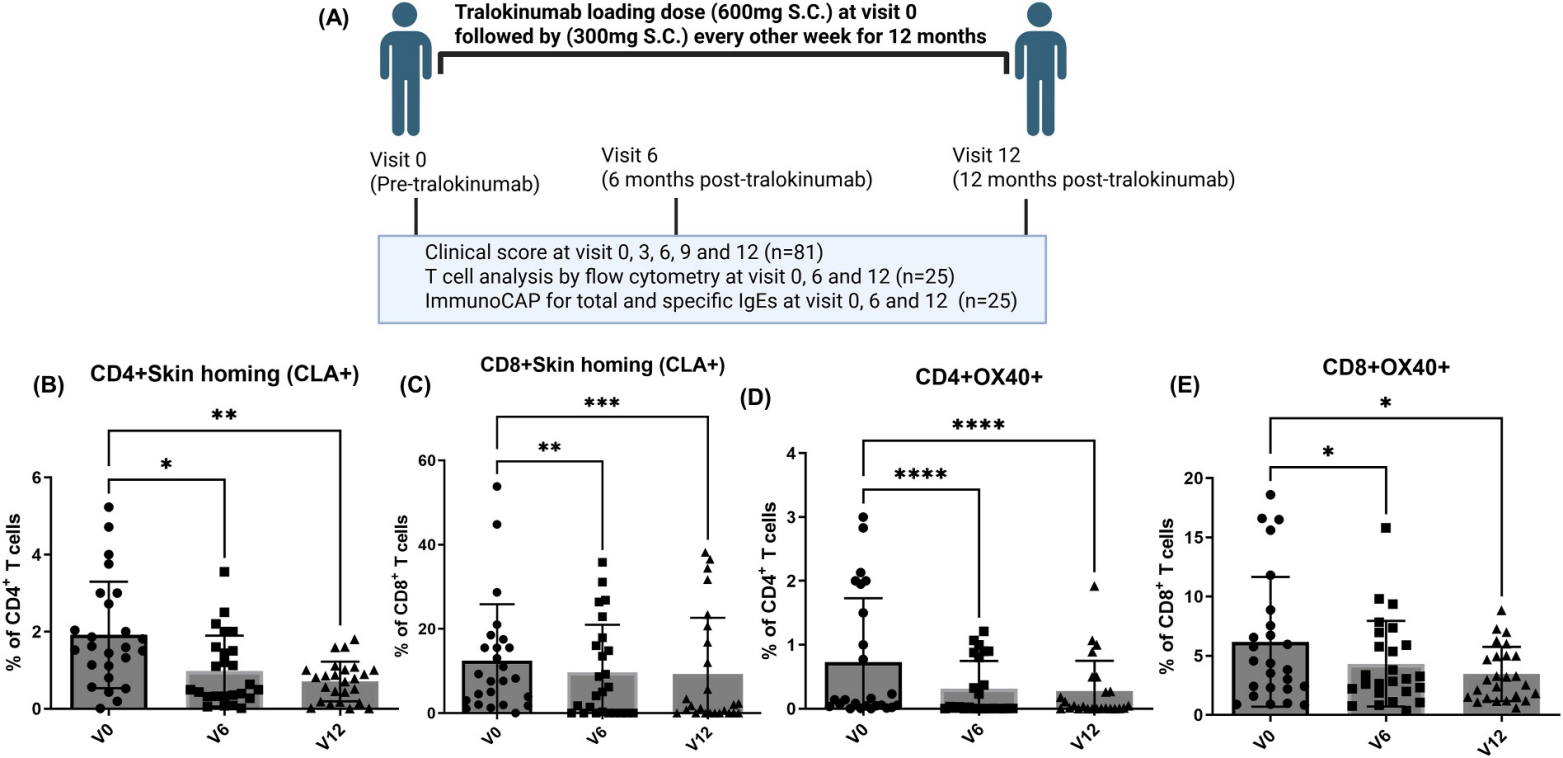
Circulating CLA^−^ and OX40^+^ CD4^+^ and CD8^+^ T cell subsets over 12 months of anti‐IL‐13 treatment. (A) Graphical depiction of the study design. Percentages of (B) CD4^+^CLA^+^, (C) CD8^+^CLA^+^, (D) CD4^+^OX40^+^, and (E) CD8^+^OX40^+^ T cells in PBMCs from patients with AD over a tralokinumab treatment period of 12 months. Data are presented as mean ± SD (*n* = 25). Statistical significance: *p < 0.05, **p < 0.01, ***p < 0.001, ****p < 0.0001.

Figure 1A illustrates the methodological framework of the recruited cohort. A total of 85 adult subjects diagnosed with moderate‐to‐severe atopic dermatitis (AD) for a minimum duration of 12 months were enrolled after providing written informed consent (ethic approval number: EA1/122/19). Disease severity was determined based on clinical criteria including body surface area (BSA) involvement of ≥ 10%, an Investigator's Global Assessment (IGA) score of at least ≥ 2, visible eczema in exposed areas, insufficient response to topical therapies or a persistent disease course. Patients meeting at least one of these indicators of moderate‐to‐severe AD were eligible for inclusion. All included patients were biologic‐ and JAK inhibitor–naive at study entry.

Participants received tralokinumab with a 600 mg subcutaneous loading dose and subsequent 300 mg biweekly, in accordance with standard clinical practice.

Clinical assessment of AD severity encompassed both patient‐reported outcomes (itch intensity and sleep disruption) and investigator‐assessed indices such as objective Scoring Atopic Dermatitis (oScorad), investigator global assessment (IGA) and body surface area (BSA) involvement [16, 17]. Due to ethical and practical limitations, not all patients who completed clinical scoring at the inclusion (*n* = 81) consented to the collection of blood samples for immunological profiling; therefore, only a subset (*n* = 25) underwent peripheral blood sampling for immunophenotyping.

### 2.2 PBMC Isolation

Peripheral blood mononuclear cells (PBMCs) were extracted from whole blood specimens utilizing density gradient centrifugation with Ficoll‐Paque (GE Healthcare), as described previously [18]. After the isolation, the cellular concentration was standardized to 2 × 10^7^ cells/mL employing Recovery Cell Culture Freezing Medium (Gibco). The resulting cell suspension was subsequently stored into cryogenic vials and preserved at −80°C for future experimental applications. The duration of storage varied from 1 to 12 months. Cell viability was routinely assessed using the trypan blue to determine the number of viable (live) and non‐viable (dead) cells. The cell viability varied from 30% to 70% live cells upon thawing.

### 2.3 Flow Cytometry

For details, please see Supporting Information S1 and methods.

### 2.4 Statistical Analysis

Statistical evaluations were conducted utilizing Prism (version 9, GraphPad Software). For the larger clinical cohort (*n* = 81), clinical outcomes were analyzed using all available observations at each study visit; due to longitudinal attrition, the Kruskal–Wallis test was employed to maximize the inclusion of all available data points. For the immunophenotyping subset (*n* = 25), where longitudinal follow‐up was more complete, we performed repeated‐measures analysis using the Friedman test followed by Dunn's post‐hoc test to account for intra‐patient variability over time. Spearman correlation was used to assess the association between T cell subsets and BSA. Correlation coefficients >  0.25 and *p* < 0.05 were used to indicate a positive correlation between T cell subsets and BSA. A *p*‐value of ≤ 0.05 was deemed to indicate statistical significance.

## 3 Results

### 3.1 Demographics

Eighty‐one moderate‐to‐severe AD patients were included and follow‐up data on demographics were available for 76, 70, 60, and 58 patients at 3, 6, 9, and 12 months. The cohort comprised 45 males (53%) and 40 females (47%), with a median age of 39 years (range: 31–53). Among 67 patients reporting disease duration, the median was 30 years (range: 21–40). Early‐onset AD (< 18 years) was noted in 52 patients, and late‐onset in 15. Of 79 patients with atopic comorbidity data, 22 (27.8%) had AD only, 28 (35.4%) had asthma, 51 (64.6%) allergic rhinoconjunctivitis, and 25 (31.6%) food allergy (Table 1).

**TABLE 1:** Clinical features, gender distribution, age, atopic comorbidities, and AD duration in the bionaive cohort.

Characteristics	Baseline (*n* = 85)
Sex, *n* (%)	
Male	45 (53)
Female	40 (47)
Age (years), median (IQR)	39 (31–53)
Atopic comorbidities^a^, *n* (%)	
Asthma bronchiale	28 (35.4)
Allergic rhinoconjunctivitis	51 (64.6)
Food allergy	25 (31.6)
AD duration (years), median (IQR)^b^	30 (21–40)
AD‐onset^b^	
≤ 18 years	52
> 18 years	15

^a^ *n* = 79 due to missing data.

^b^ *n* = 67 due to missing data.

### 3.2 Clinical Efficacy

Clinical scores (oScorad, IGA, BSA) were determined from 81, 74, 69, 58, and 58 patients at months 0, 3, 6, 9, and 12. At baseline, 17 patients had IGA ≥ 2 (20.9%), 60 patients IGA‐3 (74%), and 4 patients IGA‐4 (4.9%) (Table 2). Mean oScorad decreased from 39.3 to 15.4, and BSA from 27.8% to 5.9% over 12 months (Figure 1B,C, ****p < 0.0001). No significant changes were noted between consecutive visits. IGA 0/1 response rates improved over time, reaching 53.4% at month 12 (*p* = 0.01, Table 2).

**TABLE 2:** IGA distribution, clinical scores (oScorad, BSA, Pruritus‐VAS, DLQI) and TEWL‐values over the course of therapy.

Scores	V0 (*n* = 81)	V3 (*n* = 74)	V6 (*n* = 69)	V9 (*n* = 58)	V12 (*n* = 58)	*p*‐values^a^
IGA‐score, *n* (%)						
Clear (0)	0 (0)	3 (4)	4 (5.7)	3 (5.1)	6 (10.3)	
Almost clear (1)	0 (0)	18 (24.3)	22 (31.8)	25 (43.1)	25 (43.1)	
Mild (2)	17 (20.9)	35 (47.2)	31 (44.9)	22 (37.9)	23 (39.6)	
Moderate (3)	60 (74)	18 (24.3)	11 (15.9)	7 (12)	4 (6.8)	
Severe (4)	4 (4.9)	0 (0)	1 (1.4)	1 (1.7)	0 (0)	
oScorad, mean (SD)	39.3 (12.6)	21.1 (11.9)	20.0 (11.3)	17.5 (11.2)	15.4 (10.5)	< 0.0001
BSA, mean (SD)	27.8 (17.9)	15.3 (16.3)	10.0 (12.4)	9.4 (11.6)	5.9 (7.6)	< 0.0001
IGA0/1, *n* (%)	0	21 (28.4)	26 (37.7)	28 (48.3)	31 (53.4)	0.017^b^
Pruritus‐VAS, mean (SD)^c^	5.9 (2.2)^d^	3.8 (2.5)	4.0 (2.4)	3.5 (2.4)	3.0 (2.3)	< 0.0001
DLQI, mean (SD)^c^	12.9 (7.1)	6.5 (5.5)	6.1 (5.8)	4.4 (4.4)	4.6 (4.9)	< 0.0001
TEWL g/m^2^/h mean (SD)^e^	19 (13.7)		19.1 (15.9)		16.3 (11.7)	ns

*Note:* Shapiro–Wilk normality test applied to all parameters.

^a^ Kruskal–Wallis test.

^b^ Chi‐square test.

^c^ *n* = 77, 69, 66, 56, 51 due to missing data.

^d^ *n* = 79.

^e^ *n* = 51, 54, 48 due to missing data.

Subjective scores also improved: Pruritus‐VAS declined from 5.9 to 3.0, and DLQI from 12.9 to 4.6 by month 12 (Figure S1A–D, ****p < 0.0001), with no significant differences between post‐baseline visits. Subgroup analyses by age of AD onset (≤ 18 vs. > 18 years), sex, and atopic comorbidities showed no significant differences in oScorad, IGA or pruritus‐VAS outcomes.

### 3.3 Safety and Adverse Events

Eye manifestations were the most frequently reported adverse events, peaking at month 6 with 32.9% (23/70) of patients being affected. The primary symptoms included itching, dry eyes and conjunctivitis. Most adverse events were mild and did not require treatment discontinuation. However, four patients stopped therapy due to adverse events including recurrent pneumonia, bronchitis, dyspnea, fatigue, joint complaints and hair loss.

Herpes infections occurred in four patients: two cases of herpes zoster (including one severe ophthalmicus) and two cases of herpes keratitis. Hair loss was reported in three patients, leading to discontinuation in two; one patient continued and improved after 9 months. Adverse events by visit are summarized in Table 3.

**TABLE 3:** Adverse events in the study cohort.

Adverse events	3 months (*n* = 71)	6 months (*n* = 70)	9 months (*n* = 60)	12 months (*n* = 58)
Patients with adverse events, *n* (%)	28 (39.4)	29 (41.4)	24 (40)	20 (34.4)
Severity of adverse event, *n* (%)				
Mild	18 (64.2)	20 (68.9)	15 (62.5)	14 (70)
Moderate	7 (25)	8 (27.5)	9 (37.5)	4 (20)
Severe	3 (10.7)	1 (3.4)	0 (0)	2 (10)
Adverse events leading to treatment discontinuation^a^	2 (7.1)	1 (0)	0 (0)	1 (5)
Eye disorders	17 (60.7)	23 (79.3)	18 (75)	15 (75)
Conjunctivitis	8 (28.5)	6 (20.6)	4 (16.6)	4 (20)
Blepharitis	1 (3.5)	3 (10.3)	2 (8.3)	2 (10)
Herpes keratitis	0 (0)	0 (0)	1 (4.1)	1 (5)
Dry/itching eyes	8 (28.5)	14 (48.2)	11 (45.8)	8 (40)
Injection side reactions	8 (28.5)	6 (20.6)	4 (16.6)	1 (5)
Increased incidence of infections^b^	4 (14.2)	1 (3.4)	2 (8.3)	2 (10)
Herpes infections^c^	0 (0)	1 (3.4)	2 (8.3)	2 (10)
Pneumonia	1 (3.5)	0 (0)	0 (0)	0 (0)
Hair loss	3 (10.7)	2 (6.8)	1 (4.1)	1 (5)
Joint complaints	1 (3.5)	0 (0)	0 (0)	0 (0)
Fatigue	2 (7.1)	0 (0)	0 (0)	0 (0)
Stabbing and/or tingling sensation of skin	3 (10.7)	1 (3.4)	1 (4.1)	0 (0)
Headache	1 (3.5)	0 (0)	0 (0)	0 (0)
Acne	0 (0)	1 (3.4)	3 (12.5)	1 (5)

^a^ Pneumonia, dyspnoea, joint problems, fatigue, and hair loss.

^b^ Common cold, pneumonia, bronchitis, etc.

^c^ Herpes simplex, herpes zoster, and varicella.

### 3.4 Reduced Skin‐Homing and Activated T‐Cells in Peripheral Blood

First, we investigated the skin‐homing (CLA^+^) and activated (OX40^+^) T‐cells (Figure 1B–E). Tralokinumab treatment resulted in a marked reduction of skin‐homing (CLA^+^) and activated (OX40^+^) T‐cells. CD4^+^CLA^+^ T‐cells decreased by 48.9% (*p < 0.05) at 6 months and 63% (**p < 0.01) at 12 months (Figure 1B), while CD8^+^CLA^+^ T‐cells declined by 22.4% (**p < 0.01) and 25.8% (***p < 0.001), respectively (Figure 1C). This was accompanied by increased CD4^+^ effector memory T‐cells, with enhancement of 4.4‐fold and 4.6‐fold at visits 6 and 12 (****p < 0.0001) (Figure S2A), and a 26.6% increase in CD8^+^ effector memory T‐cells at visit 12 over baseline (*p < 0.05) (Figure S2B).

Furthermore, CD4^+^OX40^+^ T‐cells decreased by 57.3% (****p < 0.0001) and 62.4% (****p < 0.0001) at visits 6 and 12 (Figure 1D), while CD8^+^OX40^+^ cells were less severely reduced (29.9% and 44.0%, *p < 0.05) over baseline (Figure 1E). CD4^+^ EMRA cells decreased by 0.71‐fold at visit 6 and 0.66‐fold (**p < 0.01) at visit 12 (Figure S2C), while CD8^+^ EMRA and central memory cells remained stable (Figure S2D–F). The frequency of CD4^+^CLA^+^ T‐cells correlated positively with BSA, whereas CD4^+^OX40^+^ T‐cells did not (Figure S4A,B). Collectively, these data demonstrate a significant phenotypic shift in the peripheral blood, characterized by a distinct reduction in activated and skin‐homing T‐cells alongside an expansion of the effector memory pool.

### 3.5 Rebalancing of CD4^+^ T‐Cell Cytokine Responses

**FIGURE 2:**
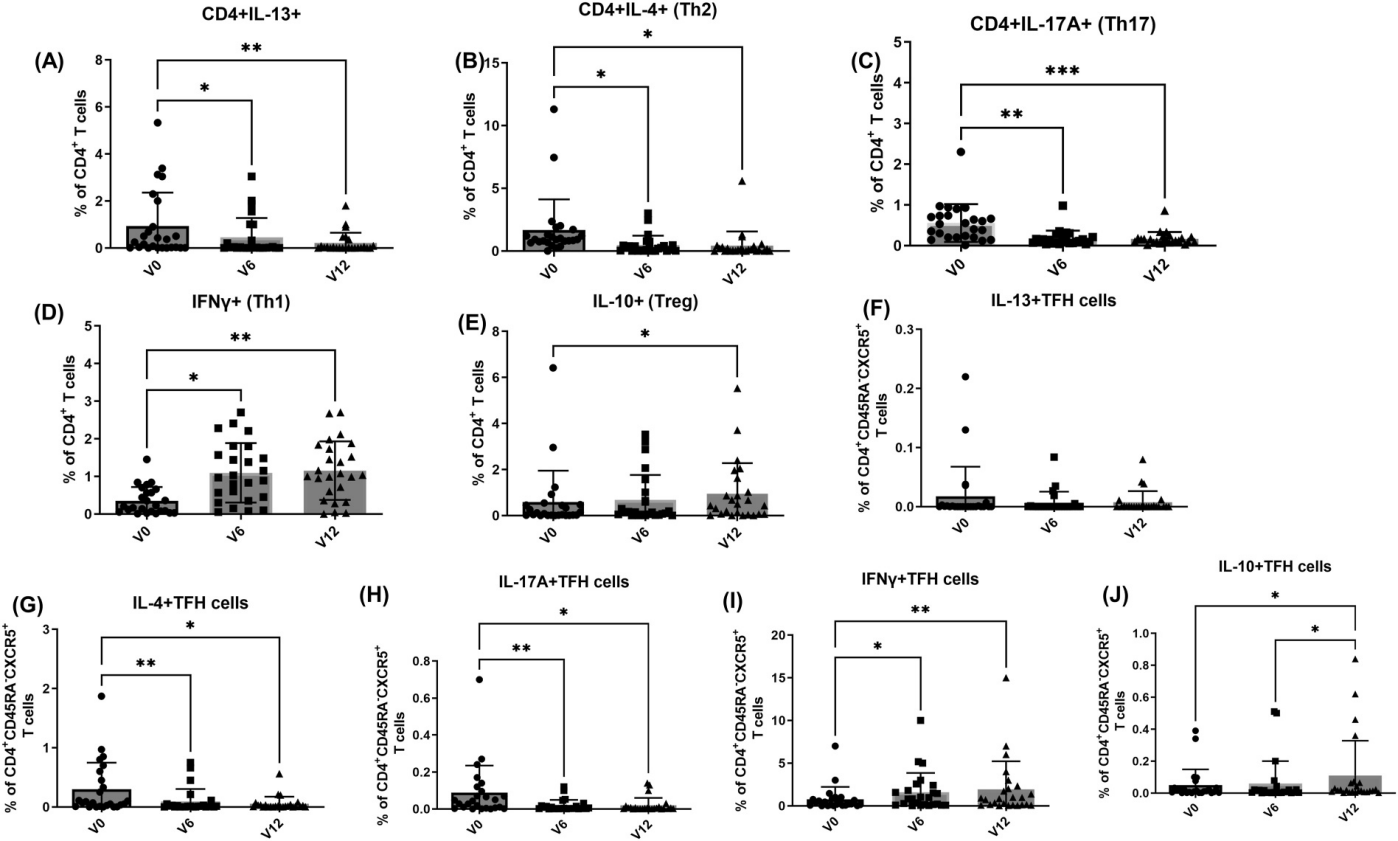
Frequencies of cytokine‐producing T cell subsets during tralokinumab treatment. Percentages of (A) CD4^+^IL‐13^+^, (B) CD4^+^IL‐4^+^ (Th2), (C) CD4^+^IL‐17A^+^ (Th17), (D) CD4^+^IFNγ^+^ (Th1), (E) CD4^+^CD25^+^CD127‐IL‐10^+^ (Treg cells), (F) CD4^+^CD45RA^−^CXCR5^+^IL‐13^+^ (IL‐13^+^TFH cells), (G) CD4^+^CD45RA^−^CXCR5^+^IL‐4^+^ (IL‐4^+^TFH cells), (H) CD4^+^CD45RA^−^CXCR5^+^IL‐17A^+^ (IL‐17A^+^ TFH cells), (I) CD4^+^CD45RA^−^CXCR5^+^IFNγ^+^ (IFNγ^+^ TFH cells), and (J) CD4^+^CD45RA^−^CXCR5^+^IL‐10^+^ (IL‐10^+^ TFH cells) T cells in PBMCs derived from AD patients upon 6 months and 12 months of tralokinumab treatment. Data are given as mean ± SD (*n* = 25). Statistical significance: *p < 0.05, **p < 0.01, ***p < 0.001.

The data of CD4^+^ T‐cell cytokine expression patterns show a shift upon tralokinumab treatment. CD4^+^IL‐13^+^ cells decreased by 58.9% (*p < 0.05) at 6 months and 70.8% (**p < 0.01) at 12 months (Figure 2A), while CD4^+^IL‐4^+^ (Th2) cells decreased by 71.2% and 72.8% at the same timepoints (*p < 0.05) (Figure 2B) from baseline. CD4^+^IL‐17A^+^ (Th17) cells also declined in a similar manner, with mean fold reductions of 0.31 (**p < 0.01) and 0.3 (***p < 0.001) at 6 and 12 months (Figure 2C).

Conversely, CD4^+^IFNγ^+^ (Th1) cells increased 3.1‐fold (*p < 0.05) and 3.2‐fold (**p < 0.01) (Figure 2D), and CD4^+^IL‐10^+^ (Treg) cells were enhanced by 62% (*p < 0.05) at 12 months from baseline (Figure 2E). In CD8^+^ T‐cells, IL‐13, IL‐4, and IL‐17A expression remained stable, while IFNγ expression increased by 16.6% (*p < 0.05) at 12 months and IL‐10 showed a transient 40.5% rise at 6 months (*p < 0.05) compared to baseline (Figure S3A–E). CD4^+^IL‐13^+^, CD4^+^IL‐4^+^, and CD4^+^IL‐17A^+^ T‐cells were positively associated with BSA and displayed a negative correlation with CD4^+^IFNγ^+^ T cells (Figure S4C–F).

Next, we analyzed cytokine profiles of follicular helper T‐cell (TFH) (CD4^+^CD45RA^−^CXCR5^+^) following tralokinumab treatment. IL‐13 TFH expression remained unchanged (Figure 2F), while IL‐4 and IL‐17A^+^ TFH cells significantly declined by mean fold decreases to 0.32 (**p < 0.01) and 0.18 (*p < 0.05) for IL‐4, and to 0.2 (**p < 0.01) and 0.22 (*p < 0.05) for IL‐17A at 6 and 12 months, respectively (Figure 2G,H).

In contrast, frequencies of IFNγ expressing TFH cells increased 2‐fold (**p < 0.01) and 2.4‐fold (**p < 0.01), also IL‐10 positive TFH cells increased by 1.2‐fold (*p < 0.05) and 2.2‐fold (*p < 0.05) at 6 and 12 months of anti‐IL‐13 therapy over baseline (Figure 2I,J). These findings suggest that tralokinumab treatment shifts the TFH cells from a Th2/Th17 to a Th1/regulatory cytokine profile. Overall, these systemic cytokine changes suggest a transition during tralokinumab therapy, characterized by a dampening of Th2 and Th17 inflammatory pathways and a concurrent enhancement of Th1 and regulatory cell signatures.

### 3.6 IgE and Eosinophil Counts Upon Anti‐IL‐13 Therapy

**FIGURE 3:**
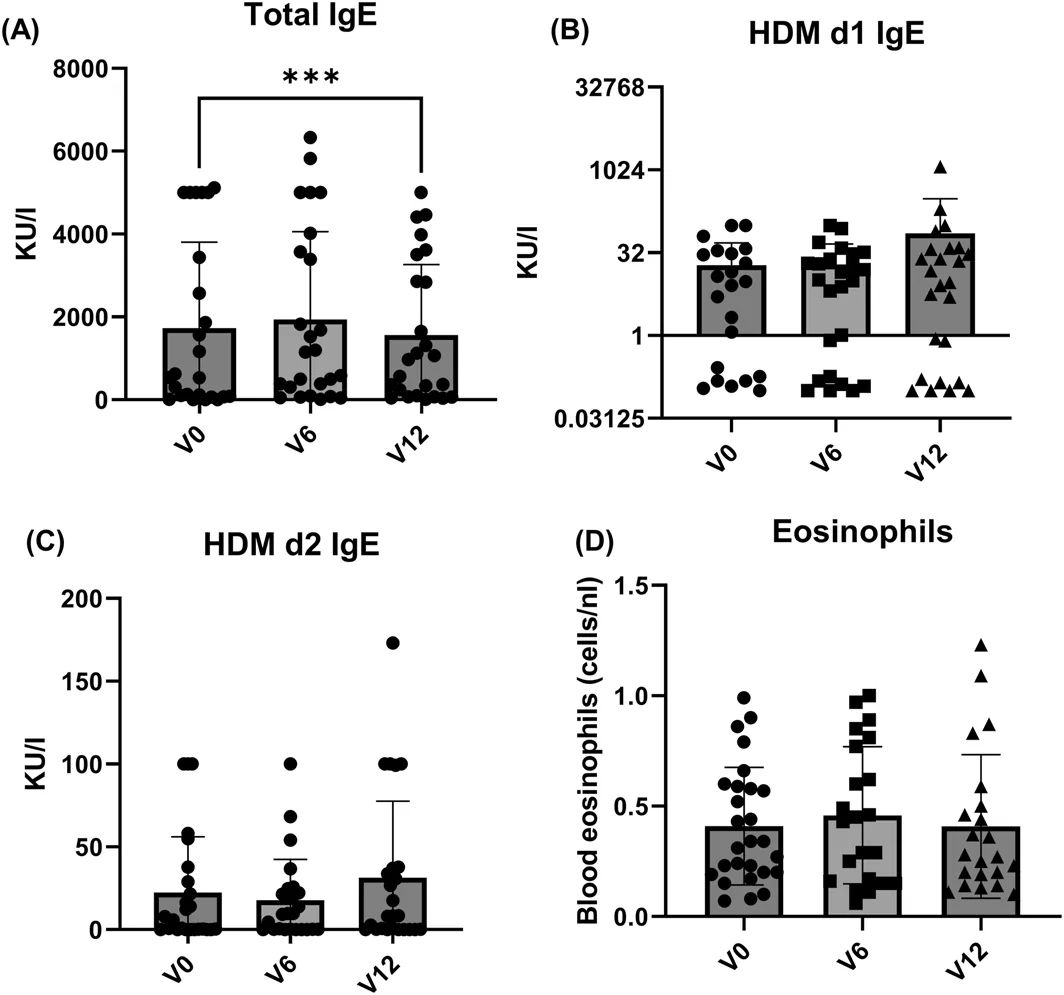
Reduction of total IgE levels, but not HDM‐specific IgE and stable eosinophil counts upon tralokinumab treatment. Serum (A) total IgE, (B) HDM *Der p1*‐specific IgE, and (C) HDM *Der p2*‐specific IgE levels *n* = 25. (D) Blood eosinophil count (*n* = 22–27) in AD patients pre‐ and post‐anti‐IL‐13 treatment over a period of 12 months. Data are presented as mean ± SD. Statistical significance: ***p < 0.001.

Total serum IgE remained stable at 6 months, but decreased by 9.7% at 12 months (***p < 0.001) after initiation of tralokinumab therapy (Figure 3A), while HDM‐specific IgE to Der p1 and Der p2 remained stable (Figure 3B,C). Peripheral blood counts, including eosinophils, leukocytes, neutrophils, and monocytes, showed no changes (Figure 3D, Figure S5A–C). However, basophils transiently increased 1.3‐fold at 6 months (*p < 0.05), and lymphocytes increased 1.1‐fold at 12 months (**p < 0.01) (Figure S5D,E).

## 4 Discussion

This study provides clinical and immunological real‐world data from a large AD cohort receiving tralokinumab for moderate‐to‐severe atopic dermatitis. Consistent with data from clinical trials [10, 11, 19, 20] as well as recently published real‐world reports [12, 13, 14, 15], we observed sustained improvements in clinical scores (oScorad, BSA, IGA) and patient‐reported outcomes (pruritus‐VAS, DLQI).

The safety profile was consistent with findings from the ECZTRA 1–3 clinical trials [10, 11]. The most frequent reported adverse event were ocular symptoms—primarily conjunctivitis, dry and itching‐eyes affecting up to 33% of patients by month 6. While higher than the conjunctivitis rate reported in ECZTRA trials (5.4%–11.2%), these events were generally mild and rarely required treatment discontinuation. The higher incidence observed in our cohort may reflect differences between clinical trial populations and routine clinical practice, where patients often present with more heterogeneous disease characteristics, pre‐existing ocular comorbidities, and are monitored under less restrictive conditions. In addition, mild ocular symptoms may be more readily documented in real‐world settings than in clinical trials, since we complemented standard adverse event reporting with patient‐reported data. Conjunctivitis typically resolved within weeks and required ongoing treatment less frequently than with dupilumab [10, 21]. Herpesvirus‐related complications occurred in 4.9% of patients, slightly higher than in the clinical trials but within the expected range for type 2 biologics [22, 23, 24]. Eosinophilia was not observed in our cohort, contrasting dupilumab‐associated findings (up to 30%) supporting the more selective safety profile upon IL‐13 targeting [25].

AD is a systemic immune disease. Therefore, we investigated the immunological consequences of tralokinumab treatment on the T cell phenotype and function, but also the IgE response. Although our immunophenotyping was based on peripheral blood, clinical improvement as reflected by oScorad and BSA indicates an improvement of cutaneous inflammation. Supporting this, recent single‐cell RNA sequencing analyses demonstrated that lesional skin and peripheral blood display similar immune signatures in patients with severe AD, particularly regarding Th2‐skewed T‐cell populations [26]. While this suggests that the immunological alterations observed in the circulation might parallel those of the skin, our findings are exploratory. Confirming whether these systemic changes directly reflect localized tissue dynamics would require paired skin biopsies. Nonetheless, our findings indicate a shift to an anti‐inflammatory T‐cell profile, including a transition toward Th1 and regulatory phenotypes and, by trend, a correspondingly decreased IgE response.

The analysis of T‐cell memory subsets revealed that upon tralokinumab treatment, a distinct T‐cell remodeling occurred in comparison with dupilumab treated patients. This was characterized by increased frequencies of CD4^+^ effector memory and reduced frequencies of TEMRA cells, while central memory subsets remained stable [18, 27]. Such pattern suggests that selective IL‐13 blockade promotes immune readiness and limits terminal differentiation, resulting in a less cytotoxic, memory‐enriched T‐cell profile, which may contribute to immunomodulatory effects consistent with disease modification.

In parallel, tralokinumab treatment resulted in a modulation of activation markers such as OX40. OX40 (CD134), a co‐stimulatory molecule promoting T‐cell activation, proliferation, survival and cytokine production [28, 29] has been linked to Th2‐driven AD. Clinical trials targeting OX40‐OX40L have been shown to improve AD symptoms [30, 31]. Moreover, data from animal models suggest that OX40 blockade reduces Th2 cytokine production, including IL‐13 [32]. In this study, tralokinumab treatment resulted in decreased frequencies of OX40^+^ CD4^+^ and CD8^+^ T‐cells, suggesting that IL‐13 may sustain OX40 expression via a cytokine‐mediated activation loop. This aligns with prior reports demonstrating elevated numbers of OX40^+^ T cells in AD skin, but also peripheral blood [33, 34].

Building upon the observed reduction of peripheral OX40^+^ T‐cells, our data show that tralokinumab treatment resulted in a modulation of cytokine profiles across multiple T helper subsets. Our findings align closely with biomarker‐based evidence of immune rebalancing under tralokinumab therapy. Previous transcriptomic and protein‐level analyses in AD patients have demonstrated that IL‐13 blockade leads to significant reductions in circulating and lesional type 2‐associated biomarkers, including CCL17/TARC, periostin, IgE, and multiple Th17 signature genes [20].

While previous studies showed that IL‐4Rα blockade via dupilumab reduces CLA^+^ T‐cells [35, 36, 37], our findings indicate that also targeted IL‐13 inhibition diminishes circulating CLA^+^CD4^+^ and CD8^+^ T‐cells.

We identified that some inflammatory T‐cell subsets showed positive association with clinical disease severity (BSA) (Figure S4A,C–E). Frequencies of CD4^+^IFNγ^+^ T‐cells were inversely correlated with BSA following treatment (Figure S4F).

In comparison with dupilumab, which inhibits both IL‐4 and IL‐13 via IL‐4Rα interference tralokinumab treatment resulted in a comparable reduction of Th2 cytokines [18, 35, 37, 38]. Despite not blocking IL‐4, IL‐13‐ inhibition appears sufficient to reduce Th2 and Th17 activity while enhancing IFNγ and IL‐10, shifting the immune environment toward an anti‐inflammatory and regulatory signature.

Our findings also suggest that a selective IL‐13 blockade extends to specialized T‐cell populations such as TFH cells, which are key regulators of humoral immunity [39]. TFH cells exhibit functional heterogeneity, with subsets producing cytokines including IL‐13, IL‐4, IL‐17A, IL‐21, IL‐10, and IFNγ [18, 40, 41, 42]. We observed a shift of the TFH cytokine profile from pro‐inflammatory Th2/Th17‐like phenotypes toward Th1 and regulatory‐associated functions over the course of treatment.

Altogether, the observed reduction in serum IgE is likely attributable to the differential modulation of T‐cell subsets and is consistent with previous reports [12, 20, 43, 44]. IgE is a key mediator of allergic inflammation, facilitating mast cell degranulation and the release of pro‐inflammatory mediators [45, 46]. The transient elevations in basophils and lymphocytes observed post‐treatment may reflect early phases of immune rebalancing triggered by selective IL‐13 blockade.

Some limitations should be considered when interpreting our findings. First, the study lacked an untreated control group, limiting direct comparisons with other therapeutic approaches. Second, patient attrition over the 12‐month follow‐up resulted in a progressive reduction in sample size. To maximize the use of available data, clinical outcomes were analyzed using all observations available at each visit rather than restricting analyses to patients with complete follow‐up. While this approach enabled inclusion of the full dataset, the decreasing number of observations at later time points should be taken into account when interpreting long‐term outcomes. Furthermore, the immunophenotyping analysis was performed on a subset of 25 patients due to ethical and practical limitations regarding blood sampling. Although this rather small sample size limits the statistical power for correlation analyses and introduces a potential selection bias, the baseline clinical characteristics of the subset did not differ significantly from the overall cohort, suggesting it remains broadly representative of the overall studied population.

In summary, this study provides real‐world evidence demonstrating that tralokinumab is an effective and well‐tolerated long‐term treatment for moderate‐to‐severe AD. Beyond clinical improvement, tralokinumab is associated with systemic T‐cell immunological changes, including the reduction of skin‐homing and activated T‐cells, Th2 and Th17 cytokines, and the increase of Th1 and other T‐cell regulatory markers. Moreover, we observed a rebalance of T‐cell memory subsets and modulation of follicular helper T‐cell profiles, contributing to normalized T cell immune environment.

Future studies with head‐to‐head trials or well‐matched cohort comparisons with dupilumab or other emerging targeted treatments (e.g., anti‐IL‐31 or anti‐TSLP) will clarify whether distinct molecular pathways are modulated by different therapeutic targets, and which immune signatures may predict optimal outcome sustainability after stopping the treatment.

## Author Contributions

**Meslina Almaci:** writing – original draft, writing – review and editing, visualization, methodology, formal analysis. **Philipp Globig:** project administration, writing – review and editing, conceptualization, writing – original draft. **Davender Redhu:** writing – review and editing, writing – original draft, methodology, formal analysis. **Margitta Worm:** conceptualization, funding acquisition, writing – original draft, writing – review and editing, supervision, resources.

## Funding

This study was funded by a research grant from LEO Pharma GmbH to M.W. and by the Deutsche Forschungsgemeinschaft (DFG, German Research Foundation) as part of the clinical research unit (CRU339): Food allergy and tolerance (FOOD@)—409525714.

## Ethics Statement

This study was approved by the university ethics committee (EA1/122/19).

## Conflicts of Interest

M.A., P.G., and D.R. declare no conflict of interest. M.W. has received grants/research funding from and/or been a consultant for: AbbVie Deutschland GmbH & Co. KG, Aimmune Therapeutics UK Limited, ALK‐Abelló Arzneimittel GmbH, Allergopharma GmbH & Co KG, Almirall Hermal GmbH, Amgen GmbH, AstraZenceca GmbH, Bayer AG, Bencard Allergy GmbH, Bioprojet Pharma, Boehringer Ingelheim Pharma GmbH &Co. KG, Bristol Myers Squibb GmbH & Co. KGaA, Galderma Laboratorium GmbH, Glaxo Smith Kline GmbH & Co. KG, Infectopharm Arzneimittel und Consilium GmbH, LEO Pharma GmbH, Lilly Deutschland GmbH, Mylan Germany GmbH (A Viatris Company), Novartis AG, Octapharm AG, Pfizer Pharma GmbH, Sanofi‐Aventis Deutschland GmbH/Genzyme Europe B. B. and Stallergenes GmbH.

## Supporting information


Supporting Information S1

**Figure S1:** Clinical response upon tralokinumab treatment. (A) Mean oSCORAD, (B) mean body surface area, (C) mean pruritus‐VAS, and (D) mean dermatology life quality index (DLQI) over time dupilumab treatment, demonstrating significant clinical improvement. Data are presented as mean ± SD (*n* = 58–81). Statistical significance: ****p < 0.0001.

**Figure S2:** Tralokinumab treatment‐induced modulation of circulating memory CD4^+^ and CD8^+^ T cell subsets over time. Frequencies of (A) CD4^+^ effector memory (CD45RA^−^CCR7^−^), (B) CD8^+^ effector memory (CD45RA^−^CCR7^−^), (C) CD4^+^ EMRA (CD45RA^+^CCR7^−^), (D) CD8^+^ EMRA (CD45RA^+^CCR7^−^), (E) CD4^+^ central memory (CD45RA^−^CCR7^+^), (E) CD8^+^ central memory T cells in PBMCs from patients with AD over a period 12 months of anti‐IL‐13 treatment. Data are presented as mean ± SD (*n* = 25). Statistical significance: *p < 0.05, **p < 0.01, ****p < 0.0001.

**Figure S3:** Anti‐IL‐13 treatment‐induced modulation of IFNg and IL‐10 producing CD8^+^ cells. Percentages of (A) CD8^+^IL‐13^+^, (B) CD8^+^IL‐4^+^, (C) CD8^+^IL‐17A^+^, (D) CD8^+^IFNγ^+^, and (E) CD8^+^IL‐10^+^ T cells in PBMCs from patients with AD over a period 12 months of anti‐IL‐13 treatment. Data are presented as mean ± SD (*n* = 25). Statistical significance: *p < 0.05.

**Figure S4:** Correlation of CLA^+^, OX40^+^, IL‐13^+^, IL‐4^+^, IL‐17A^+^, and IFNγ^+^ CD4^+^ T cell frequencies with BSA across all time points demonstrate a relationship to the BSA score. Repeated measures correlation was used to evaluate the overall intra‐individual association between clinical measurements (BSA) and T cell subset frequencies. Because each patient contributes data from multiple visits (V0, V6 and V12), the repeated measures correlation method fits a separate but parallel regression line for each individual. CLA^+^, OX40^+^, IL‐13^+^, IL‐4^+^, IL‐17A^+^, and IFNγ^+^ CD4^+^ T cell frequencies had correlation coefficient of 0.474, 0.1833. 0.275, 0.304, 0.504, and −0.267, respectively, compared with repeated measurement of the BSA score.

**Figure S5:** Anti‐IL13 treatment did not alter peripheral blood cells except basophils and lymphocytes in AD patients. Blood count of (A) leukocytes (*n* = 27–31), (B) neutrophils (*n* = 22–27), (C) monocytes (*n* = 20–27), (D) basophils (*n* = 17), and (E) lymphocytes (*n* = 17) in AD patients pre and post anti‐IL‐13 therapy. Data were presented as mean ± SD. Statistical significance: *p < 0.05, **p < 0.01.

## Data Availability

Data will be provided on a reasonable request from the corresponding author.
