# Supplementary material for: Real‐World Efficacy and Systemic Immune Modulation Upon Tralokinumab Treatment in Atopic Dermatitis
Source: Clin Transl Allergy. 2026 Sep 6;16(9):e70200. doi: 10.1002/clt2.70200 (PMC13547590; doi:10.1002/clt2.70200)
Supplement: Supplementary file 1 [file CLT2-16-e70200-s002.docx]

**Supplementary method:**

**Flow cytometry**

Cryopreserved peripheral blood mononuclear cells (PBMCs) were thawed in a 37 °C water bath, followed by immediate washing and resuspension in Roswell Park Memorial Institute (RPMI) 1640 medium (Gibco), with 10% fetal bovine serum (FBS; Bio&Sell, Lot# BS.338199D) and 1% penicillin-streptomycin (Gibco, Lot# 2087433).

Subsequently, 1 × 10⁶ PBMCs were plated per well into round-bottom 96-well plates and allowed to incubate overnight. The next day, the cells underwent stimulation for 4 hours using a combination of phorbol 12-myristate 13-acetate (25 ng/mL; Sigma-Aldrich) and ionomycin (2 µg/mL; Sigma-Aldrich) in conjunction with Brefeldin A (10 μg/mL; Sigma-Aldrich).

Cell viability was evaluated utilizing the Live/DEAD™ Fixable Near-IR Dead Cell Stain Kit (Invitrogen). For the analysis of surface markers, cells were incubated with a panel of T cell-specific antibodies (as listed in Table S1) for 15 minutes at room temperature in FACS buffer (0.1% bovine serum albumin, Serva Electrophoresis GmbH, Lot# 2087433 in phosphate-buffered saline).

To evaluate intracellular cytokine production, cells were fixed and permeabilized employing fixation and permeabilization buffers (eBioscience, Invitrogen), followed by staining with cytokine-specific antibodies (as specified in Table S2) for 30 minutes in FACS buffer. Data acquisition was conducted on a CytoFLEX flow cytometer (Beckman Coulter GmbH), and the data were analyzed using FlowJo software.

**Supplementary figure legends:**

S Fig. 1 **Clinical response upon tralokinumab treatment.** (A) mean oSCORAD (B) mean body surface area (C) mean pruritus-VAS and (D) mean dermatology life quality index (DLQI) over time dupilumab treatment, demonstrating significant clinical improvement. Data are presented as mean ± SD (*n* = 58-81). Statistical significance: ****p< 0.0001.

S Fig. 2 **Tralokinumab treatment-induced modulation of circulating memory CD4+ and CD8⁺ T cell subsets over time.** Frequencies of (A) CD4+ effector memory (CD45RA-CCR7-) (B) CD8+ effector memory (CD45RA^-^ CCR7^-^) (C) CD4+ EMRA (CD45RA^+^ CCR7^-^) **(D) CD8+ EMRA** (CD45RA^+^ CCR7^-^) **(E) CD4+ central memory** (CD45RA^-^ CCR7^+^) (E) CD8+ central memory T cells in PBMCs from patients with AD over a period 12 months of anti-IL-13 treatment. Data are presented as mean ± SD (*n* = 25). Statistical significance: *p< 0.05, **p< 0.01, **** p< 0.0001.

S Fig. 3 **Anti-IL-13 treatment-induced modulation of IFNg and IL-10 producing CD8+ cells.** Percentages of (A) CD8+IL-13+ (B) CD8+IL-4+ (C) CD8+IL-17A+ (D) CD8+IFNγ+ and (E) CD8+IL-10+ T cells in PBMCs from patients with AD over a period 12 months of anti-IL-13 treatment. Data are presented as mean ± SD (*n* = 25). Statistical significance: *p< 0.05.

S Fig. 4 **Correlation of CLA+, OX40+, IL-13+, IL-4+, IL-17A+ and IFNγ+ CD4+ T cell frequencies with BSA across all time points demonstrate a relationship to the BSA score.** Repeated measures correlation was used to evaluate the overall intra-individual association between clinical measurements (BSA) and T cell subset frequencies. Because each patient contributes data from multiple visits (V0, V6 and V12), the repeated measures correlation method fits a separate but parallel regression line for each individual. CLA+, OX40+, IL-13+, IL-4+, IL-17A+ and IFNγ+ CD4+ T cell frequencies had correlation coefficient of 0.474, 0.1833. 0.275, 0.304, 0.504 and -0.267 respectively, compared with repeated measurement of the BSA score.

S Fig. 5 **Anti-IL13 treatment did not alter peripheral blood cells except basophils and lymphocytes in AD patients.** Blood count of (A) leukocytes (*n* = 27-31) (B) neutrophils (*n* = 22-27) (C) monocytes (*n* = 20-27) (D) basophils (*n* = 17) and (E) lymphocytes (*n* = 17) in AD patients pre and post anti-IL-13 therapy. Data were presented as mean ± SD. Statistical significance: *p< 0.05, **p< 0.01.

Table S1: Antibodies used for surface staining of T cells

| **Antibody** | **Fluorochrome** | **Supplier** | **Clone** | **Dilution** |
| --- | --- | --- | --- | --- |
| CXCR3 | BV650 | BD OptiBuild | 1C6/CXCR3 | 1:20 |
| CCR6 | BV785 | Biolegend | G04E3 | 1:20 |
| CXCR5 | PE-Dazzle | Biolegend | L138D7 | 1:100 |
| CD25 | ECD | Beckman Coulter | BE.49.9 | 1:10 |
| CD45RA | FITC | Biolegend | HI100 | 1:20 |
| OX40(CD134) | Per/CP-Cy5.5 | Biolegend | ACT35 | 1:20 |
| CD127 | PE-Cy5 | Biolegend | A019D5 | 1:20 |
| CD3 | AF700 | Biolegend | OKT3 | 1:20 |
| CCR7 | APC-Cy7 | Biolegend | G043H7 | 1:20 |
| ViaKrome808-Live/Dead | Zombie NIR | Beckman coulter | N/A | 1:50 |
| CD8 | BV421 | Biolegend | SK1 | 1:20 |
| CD4 | Spark UV387 | Biolegend | SK3 | 1:20 |
| CLA(CD162) | BUV496 | BD BioScience | KPL-1 | 1:500 |

Table S2: Antibodies used for intracellular staining of T cells

| **Antibody** | **Fluorochrome** | **Supplier** | **Clone** | **Dilution** |
| --- | --- | --- | --- | --- |
| IFNγ | BUV661 | Invitrogen | 4S.B3 | 1:20 |
| IL-2 | BV510 | BD BioScience | 5344.111 | 1:20 |
| IL-17 | BV605 | Biolegend | BL168 | 1:20 |
| IL-4 | PE | Biolegend | MP4-25D2 | 1:20 |
| IL-9 | PE-Cy5.5 | Biolegend | MH9A4 | 1:20 |
| IL-13 | PE-Cy7 | Biolegend | JES10-5A2 | 1:20 |
| IL-10 | APC, AF647 | Biolegend | JES3-19F1 | 1:20 |

Table S2: Antibodies and live/dead (Zombie) dye used for functional characterisation of T cells.
